# Effects of tree species diversity and stand structure on carbon stocks of homestead forests in Maheshkhali Island, Southern Bangladesh

**DOI:** 10.1186/s13021-021-00175-6

**Published:** 2021-04-28

**Authors:** Tarit Kumar Baul, Avinanda Chakraborty, Rajasree Nandi, Mohammed Mohiuddin, Antti Kilpeläinen, Taslima Sultana

**Affiliations:** 1 Institute of Forestry and Environmental Sciences, University of Chittagong, Chittagong, 4331 Bangladesh; 2 Faculty of Science and Forestry, School of Forest Sciences, University of Eastern Finland (UEF), P.O. Box 111, 80101 Joensuu, Finland

**Keywords:** Biomass, Biodiversity, Carbon stock, Homestead forests, Litterfall, REDD, Soil, Tree species

## Abstract

**Background:**

The homestead forests of Bangladesh occupy 0.27 million hectares (10% of the total forested area) and have potential to store carbon (C) and conserve biodiversity. Small scale forestry practices, however, are lacking reliable estimation of C stocks and tree species diversity. This may hinder successful implementation of REDD + and similar mechanisms as they concentrate on large-scale forests. This study aimed to estimate the above- and below-ground carbon stocks in homestead forests of Maheshkhali Island in Bangladesh and how tree species diversity and stand structural variation affect these C stocks. We randomly surveyed a total of 239 homestead forests in the hillside, beachside, and inland in 2019.

**Results:**

Tree biomass C stocks were 48–67% greater in the inland and hillside forests than in the beachside due to significantly greater stand density, basal area, tree diameter. In total we found 52 tree species, but most abundant species in the inland and hillside forests, *Mangifera indica*, *Samanea saman*, and *Artocarpus heterophyllus* stored the most C in tree biomass. Greater tree species richness and diversity index in the inland and hillside forests indicated greater above- and below-ground tree biomass C stocks. An increase in tree species richness and diversity index by one unit was found to increase the tree biomass C stock by 22 and 30 Mg C ha^−1^, respectively. The total soil C stock was also affected by tree species diversity, stand density, and their interaction with soil properties. Total soil C stocks were greatest (51 Mg ha^−1^) in the inland forests, having also the greatest stand density and tree species richness. C stock in soil surface was greatest in the hillside forests due to the greatest litterfall, but the average share of litterfall from the total biomass C was only 0.1%.

**Conclusions:**

Homestead forest ecosystems could store 96 Mg C ha^−1^ in total, which can contribute to climate change mitigation by generating C credits for small-scale homestead forests owners. Above- and below-ground tree biomass C stocks were found to correlate with tree species diversity, which may also contribute to biodiversity conservation in the REDD + in Bangladesh and countries alike.

## Background

A gradual increase in the global emissions of carbon dioxide (CO_2_) and consequent temperature increase has become a major concern to work on emissions mitigation [1–3]. Tropical forests play an important role in removing atmospheric CO_2_ as they store one fourth of the global terrestrial carbon [4–6]. In addition, tropical forests support at least two-thirds of the world’s biodiversity (e.g., [7]) and have 50% of all known plant species, while their coverage from the total land area of the Earth is about 12% [8].

In Bangladesh, CO_2_ emissions over the time are rapidly increasing, for example, 609% in 2017 (78 Mt of CO_2_) compared to that of 1990 (11 Mt of CO_2_) due to an increase in energy consumption [9, 10]. Bangladesh´s contribution to global emissions is very low; however, its carbon rich forest ecosystems are highly affected by land-use change and adverse impacts of climate change [11], such as changes in precipitation and global sea level rise. Bangladesh has forest areas of about 2.53 million hectares, representing 17.5% of the total land area [12, 13]. These tropical forests are consisted of hill, mangrove, sal forests, and coastal mangrove plantations, with semi-evergreen and deciduous tree coverage. Natural hill and sal forests are being degraded due to illicit felling, shifting cultivation, and conversion to other land uses [14, 15]. Apart from these natural and planted forests, tree outside forest (TOF) including homestead forests, roadside plantation is booming in Bangladesh [13].

The homestead forests of Bangladesh occupy 0.27 million hectares land area, representing 10% and 2% of the total forested land area and total land area, respectively [13, 16] and have potential to store carbon in biomass [17, 18]. The contribution of homestead forests to rural economy is second to agriculture and these forests provide people’s daily needs. The homestead forests of Bangladesh supply 70% of total timber and 90% of fuelwood and bamboo demand in the country [13, 19] and thus, release pressure on natural forests. The homestead forests can be characterized as well-established land use systems for sustenance and conservation of biodiversity [20], which are maintained by at least 20 million households. Since homestead forestry is practiced primarily for supplying daily necessities as a livelihood option, understanding the carbon stocks of the homestead forests is required to address their potential in climate change mitigation. The homestead forests in Bangladesh are in pressure due to fragmentation of landholdings [21] and not under the national forest management plan.

A few researches have estimated above-ground forest carbon stocks in Bangladesh. Carbon stocks were found to vary with land uses, including mangrove and coastal (99 Mg C ha^−1^; [22]), protected contiguous and fragmented (34–53 Mg C ha^−1^; [15]), bamboo (53 Mg C ha^−1^; [23]), hill (103 Mg C ha^−1^; [16]), and homestead (53 Mg C ha^−1^; [17]) forests. These carbon stocks have been found to be dependent on the stand structure (e.g., tree height, DBH, density, basal area) [24, 25] and tree species [26, 27], and stands with fast-growing tropical tree species having the highest forest carbon stocks (201 Mg C ha^−1^; [28]). Tree species diversity may increase above-ground biomass carbon stocks of tropical forests [29, 30].

The carbon stock in litterfall is only a small fraction relative to above-ground biomass carbon in forest ecosystems [31], but this needs to be studied when estimating carbon dynamics among pools [32]. A balance between accumulation and decay of litter controls the accumulation of organic matter in an ecosystem [33]. Within the same climate, forest and tree species types are the main drivers of the litterfall, e.g., in mixed species natural forests and planted forests [34]. Litterfall is also affected by the management such as tree harvest and pruning [35–37]. Research on carbon stock in litterfall has been very scarce in the homestead forest of Bangladesh, and generally in tropical and temperate forests [38].

In Bangladesh, soil carbon concentration has been found variable (1–16 mg g^−1^), mainly responsible to the site and depth in soil [39, 40]. Soil carbon stocks estimated in Bangladesh were 23 Mg C ha^−1^ in semievergreen [41], 34 Mg C ha^−1^ in mangrove [22], and 59 Mg C ha^−1^ in deciduous [42] forests. Earlier, changes in stand structure and litter quality have found to modify the soil carbon dynamics in agroforestry ecosystems and also in tropical homestead forests [43–46]. The dynamics are also influenced by microclimatic and edaphic conditions [47–49] and they vary in space and time [50]. For instance, tree size, stand density, and species richness positively affected soil carbon in tropical forests [51, 52]. While significant advances in estimating the carbon balance of forests have been attained, there are still critical uncertainties in the magnitude of soil carbon stocks [37].

The United Nations Framework Convention on Climate Change (UNFCCC) introduced mitigation instruments, clean development mechanism (CDM) and reducing emissions from deforestation and forest degradation and conservation and enhancement of forest carbon stock with sustainable management (REDD +) [2, 53, 54]. REDD aims to maintain carbon stock within tropical forests while conserving biodiversity [55]. However, there is uncertainty in biodiversity provision within REDD [56] and lack of clear understanding on interactions of carbon dynamics and biodiversity [57]. A positive corelation between estimated biomass carbon and biodiversity exits globally [58], but there is spatial variation [57], which makes REDD initiative complex at regional or sub-national scales. Moreover, inadequate data on carbon stock in local forests (Baccini et al. [59]) and the lack of reliable estimation of tropical forests carbon stocks may hinder the effective implementation of REDD + and similar mechanisms [60]. More importantly, REDD, or derivative REDD + , takes only large-scale forests into account, while ignoring the small-scale forests, such as homestead forests [61]. The evidence of carbon sequestration potential of TOF can be significant for small-scale forest landowners or households in developing countries, such as Bangladesh, and it can be also used to support international treaties of the Paris agreement 2015 and the Kyoto Protocol 1997. In addition to the natural and planted forests managed by Bangladesh Forest Department, the estimation of the carbon stocks in homestead forests is imperative for investigating their potentials for carbon enhancement and credits [61, 62].

Under this circumstance, the study about homestead forests for estimation of carbon stocks would be the scientific-based information for the policymakers and scientists with a view to support climate change mitigation efforts. Our study aims to estimate the carbon stocks in homestead forest ecosystems (trees, litterfalls, and soil) of Maheshkhali Island under Cox’s Bazar District in Bangladesh and how tree species diversity and stand structural variation affect these carbon stocks.

## Material and methods

### Study area

**Fig. 1:**
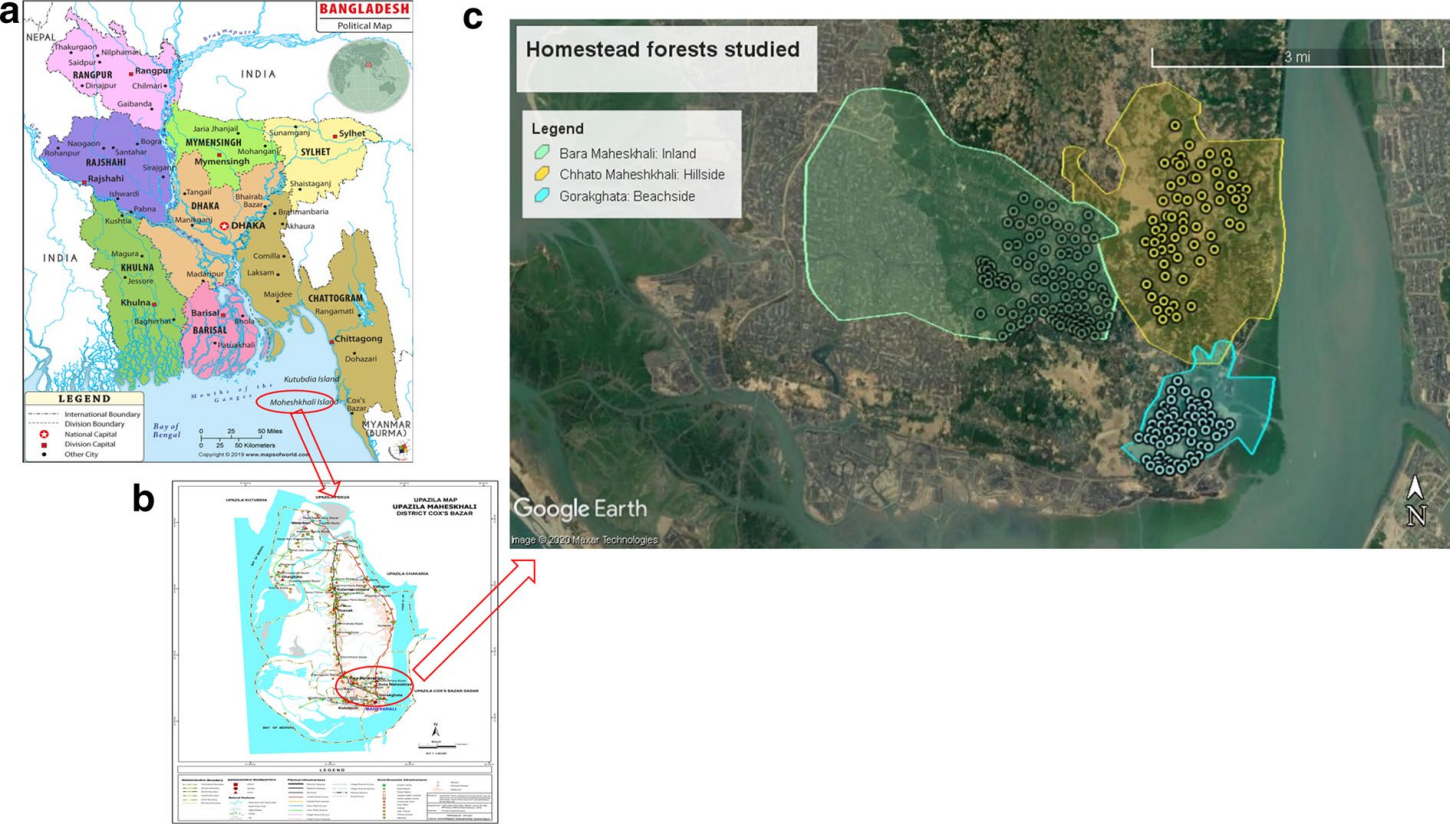
Map of (**a**) Bangladesh and (**b**) Maheshkhali Island showing three categorized study sites with (**c**) sampling points of homestead forests

The study was conducted in homestead forests of Maheshkhali Island under Cox’s Bazar District, a coastal area of Bangladesh (Fig. 1), emerged as most vulnerable to climate change impacts [11]. Maheshkhali island is the only hilly island with complex geological system on the eastern cliff coast of Bangladesh [63], located between 21°28′ and 21°46′N latitude and 91°51′ and 91°59′E longitude [64]. It occupies an area of 362.18 km^2^, with a total of 33,287 households [65]. The island has a moist tropical climate with a long wet season (April–October) and a relatively short dry season (November–March). The mean annual precipitation, temperature, and relative humidity are 3627 mm, 25.7 °C, and 70–90%, respectively [66]. This region is prone to cyclonic storms, tidal surges, and flood due to proximity to the Bay of Bengal, a source of cyclones, usually occurring during April–May and October–November.

The island has four subdivisions including active, young, and old coastal plain, and hilly areas with piedmont plain. Geological deposition of sedimentation forms landmasses [63]. Maheshkhali with an accretion rate of 1.2 sq. km. per year since 1972 formed huge landmasses in the southwest coastal plain (e.g., Bara Maheshkhali) and western coastal plain (e.g., Gorakghata) [67], contributing to the land use and land cover changes. The major land uses include salt cultivation, agricultural land, hill forests, and coastal forests, which have been changed markedly since 1972. Expansion of salt fields caused a decline in the agricultural land at an average rate of 14.5 ha per year, and extensive and illegal hill cutting for settlement, betel leaf cultivation, and unpanned development caused reduced hill forests at an average rate of 90 ha per year [64]. Shrimp cultivation, another type of land use threatened the coastal forests, to some extent, especially old coastal zone [64].

Bangladesh Forest Department manages its hill and coastal forests consisting of mangrove plantations of 534.54 ha and 8129.9 ha and non-mangrove plantations of 2667.3 sq. km and 232.66 sq. km, under two range,^1^ Maheshkhali and Gorokghata, respectively [68]. Forest covers several hills of up to 23 m and the low-laying valleys. Soils of the forest vary from clay to sandy loam and to some extent yellowish red sandy clay [69]. Peoples` dependence on hill forests were significant for collecting fuelwood, house and boat making materials, traditional medicine, and non-timber forest products such as bamboo, honey, fodder etc. However, from the time immemorial, this overexploitation of the resource declined in biodiversity in the area [70]. This situation made the individuals for taking care and managing homestead forests very carefully for their protection from coastal storms, surges, floods. Homestead forests are managed by owners themselves.

### Reconnaissance survey

Before starting the data collection, three initial field visits for reconnaissance survey were made to get an overview of the study area in February 2019. This included observation of general conditions including geographical location, physiography, hill and coastal forests, and existence of homestead forests along the hillside, beachside, and in the inland of the area. We categorized three Unions^2^ Chhato Maheshkhali (recently converted as municipality), Gorokghata, and Bara Maheshkhali as the hillside, beachside, and inland, respectively, under Maheshkhali sadar Upazila according to the geographic location (Fig. 1). Settlement was assumed to be associated with the homestead forests in the three sites. Settlement is an important type of land use, and the population density was relatively higher in the hillside (Chhato Maheshkhali), beachside (Gorokghata), and inland (Bara Maheshkhali) compared to the northwestern part of the island [71]. From the key informant (KI) interview with the Chairman of the Union in February 2019 it was known that some of the settlements started in the foot of the hills under the hillside by human intervention in modifying slope of the hills. Since after the loss of lives in 1991 cyclone, people started migrating from Gorakghata to other places [67]. Settlement started earlier in the inland site and population was relatively higher [71]. The homestead forests of these three sites were also assumed to represent the same kind of ecotype. We hypothesized that carbon stocks of the homestead forests differ from each other among the three sites.

The researchers collected relevant data of villages, number of households, and homestead forests of each village from the respective administrative (Union Parishad) offices. Additionally, we interviewed the Chairman of these three Unions as key informants to gather knowledge about the study sites and as well as inform them about the purpose of the study.

### Sample selection and woody vegetation measurement in homestead forests

The sampling procedure followed from Upazila to Union, Union to village, village to homestead forests of the households. From the lists of the number of households provided by the office, with a sampling intensity of 5% as accepted by the United Nations [72], a total of 239 homestead forests were determined. Then, based on the total number of households in each of the three sites, 67, 69, and 103 homestead forests from hillside, beachside, and inland, were randomly allocated for the study in Maheshkhali sadar Upazila in 2019 (from February to April). The mean area of the studied homestead forests in these three sites were 0.02, 0.01, and 0.02 ha per household.

Each of the homestead forests was divided into quadrats (5 m × 5 m) based on the area and the direction from the dwelling. The surveyed data were recorded which included all woody plants identification, with measurement of height (m), diameter at breast height (DBH, cm) and the area of the homestead forests. The owners of the homestead forests helped in identification with local name, and in few cases, herbarium was prepared to ensure the identification with scientific names. The height measurement was made by rangefinder and DBH by diameter tape. The coordinates of each point of sample collections was recorded by using GPS. Herbs and shrubs were not considered as 98% of total forest biomass consists of tree biomass; they may be ignored in estimation of carbon [73]. Homestead forests are well managed and therefore, are usually free from herbs and lianas.

### Soil and litterfall sampling for estimating C stock

A sampling of the litterfall was made in 4 points wherever available for each of the three different sites in 2019 (from February to April), thus making a total of 12 (3 × 4 = 12) samples. All litterfalls at each point of an area of 1 m^2^ (1 m × 1 m) was collected using a metallic frame. A pit of 30 cm depth, under the litterfall layer sampling point, was dug by using a soil auger and mineral soil samples were collected at 10, 20, and 30 cm depths. This procedure was followed for four samples consisting of 12 (4 × 3 depths = 12) subsamples for each of the three different sites, thus making a total of (12 × 3 = 36) subsamples. Accordingly, following the same procedure, 36 unaltered soil subsamples were collected using a core (volume 100 cm^3^) to measure bulk density (BD) at the same three depths in each point, following Blake [74].

### Data analyses

#### Estimation of tree (above- and below-ground) biomass, and density and basal area of stands

Above-ground biomass (AGB) was estimated by converting tree data into biomass using allometric Eqs. (1), (2), (3), and (4) for tropical trees, *Cocos nucifera*, *Areca catechu*, and *Phoenix dactylifera*, respectively (Table 1) [60, 75–77]. Below-ground tree biomass (BGB) was estimated as 15% of AGB [78]. Tree total biomass (TB) was the summing up of AGB and BGB. Finally, total carbon stock (Mg ha^−1^) was estimated as carbon content is assumed to be 50% of dry TB [79]. To estimate AGB, wood density (g cm^−3^), a required variable, which was collected from Bangladesh Forest Research Institute (BFRI) [80]. For those not found in BFRI publications we used global wood density database [81, 82]. Additionally, species level carbon was also estimated for most frequent tree species and expressed in kilogram (kg) carbon per individual across three sites. Stand density (individual ha^−1^) and basal area BA (m^2^ ha^−1^) were estimated (Eqs. 5–7). Mean values of tree biomass, density, and BA were compared among three different homestead forest sites.

**Table 1:** Equations used in analyses of data

No.	Equation	Reference
1	$AGB \left( {kg} \right) = 0.0673 \times ({\uprho }D^{2} H)^{0.976}$, where AGB above ground biomass (kg), ρ wood density (g cm^−3^), D and H are tree DBH (cm) and height (m), respectively	Chave et.al. [60]
2	AGB (kg) = 4.5 + (7.7 × H)	Hairiah [75]
3	AGB (kg) = 10 + 6.4 H	Frangi and Lugo [76]
4	AGB (kg) = − 3.956 × H^2^ + (55.247 × H) − 2.0342	Issa et al. [77]
5	Stand density (individual ha^−1^) = $\frac{n}{A} ,$ where, A an area of the homestead forest (ha)	Shukla and Chandel [83]
6	Basal area, BA (m^2^ tree^−1^) = $\frac{{\pi \left( {D \times 0.01} \right)}}{4}^{2}$	Shukla and Chandel [83]
7	BA (m^2^ ha^−1^)$= \frac{\Sigma BA}{{A \left( {ha} \right)}}$	
8	LOI % = W_1_/W_2_ × 100, where, W_1_ is loss in weight (g), W_2_ weight of oven dry soil (g), and LOI is loss on ignition	Ball 1964 [84]
9	SOC % = 0.47 × (% LOI – 1.87), where SOC denotes soil organic carbon	Ball 1964 [84]
10	SOC stock (Mg ha^−1^) = SOC % × BD × SD, where BD bulk density of soil (g cm^−3^) and SD soil depth (cm)	Pearson et al. [85]
11	$Dry mass\;of\;the\;litter\;sample\;\left( {DM, g} \right) = \frac{Dry\;mass\;of\;subsample}{{Fresh\,mass \;of\;subsample}} \times Fresh\,mass \;of\;the\;sample$	Pearson et al. [87]
12	$Litter\;DM\;per\;unit\;area\;\left( {Mg ha^{ - 1} } \right) = \frac{DM \;\left( g \right)}{{Sampling\;frame\,area\; \left( {cm^{2} } \right)}} \times 100$	Pearson et al. [87]
13	$Margalef\;Index\; = \frac{{\left( {N - 1} \right)}}{\ln \left( n \right)}$, where N is the total number of species and n is the total number of individuals of all species	Margalef [89]
14	Shannon–Wiener index, ${\text{H}} = \sum {\text{piln}}\left( {pi} \right)$, where pi is the ratio of S to n in a homestead forest. S is the individuals of each species in a homestead forest	Michael [90]
15	${\text{Frequency }}\;\left( {\text{F}} \right) = \frac{Number \;of\; homestead\; forests\;in\;which\;particular\;species\;occurs }{{Total\;number\;of\;homestead\;forests\;studied}}$	Shukla and Chandel [83]
16	${\text{Relativefrequency}},\,{\text{ RF }}\left( {\%} \right) = {\frac{{{\text{Fi}}}}{{{{\Sigma Fi}}}}}$ × 100, where Fi is the frequency of a species in ith homestead forest (i = 1, 2, 3……..)	Dallmeier et al. [91]
17	$Relative\,density, RD \left( \% \right) = \frac{S}{ n}$ × 100	Dallmeier et al. [91]

#### Laboratory analysis and estimation of carbon of litterfall and mineral soil and bulk density (BD)

To estimate soil organic carbon (SOC), soils were oven-dried at 105 °C for 72 h. After cleaning, washed silica crucibles were dried in an oven at 105 °C for half an hour and cooled in desiccators, and then mass was taken. Oven-dried soils were ground by pestle mortar and then exactly 5 g of grind soils were kept in silica crucibles and reweighed by an electric balance. The crucibles with soil were then transferred to an electric muffle furnace for igniting at 850 °C for one and a half hour. Then crucibles with soils were cooled in the desiccator and reweighed to determine the percent loss of ignition LOI (%), from which, SOC (%) was calculated (Eqs. 8 and 9). C stocks in mineral soil at three depths were calculated using BD (g cm^−3^) (Eq. 10) and expressed in Mg ha^−1^ for three different sites (Table 1). We calculated soil BD as the quotient between the dry mass of the fine fraction in the core segment and volume of the cylinder [86].

Regarding the estimation of biomass of litterfall, after taking the fresh mass of the original samples collected from each point of litter collection, adequate subsamples from the weighed original sample were made and labelled. In each plot, the number of original samples was four and subsamples three to five, depending on the wet masses of the original samples. The wet masses of all the subsamples were measured and recorded. Subsamples were oven-dried at 65 °C until reaching a constant mass and dried masses were recorded. Then, the dry mass of the original sample from the wet to dry ratio of the subsamples was estimated (Table 1; Eqs. 11 and 12). The carbon concentration was considered to be 50% of the dry mass of litter [88]. The process was repeated for all 12 original samples collected from homestead forests across three different sites. Carbon stocks in litterfall were calculated and expressed in Mg C ha^−1^ for three different sites. These carbon concentrations and stocks of litterfall and soils were compared among three different homestead forest sites.

#### Estimation of tree species richness, diversity and relative frequency and relative density

Tree species richness (Margalef index) and diversity (Shannon-Weiner Index, H) were estimated according to Eqs. 13 and 14 (Table 1). The greater value of indices of diversity indicates greater species richness and diversity in an area. In addition, the relative frequency of occurrence (RF %) and relative density (RD %) for species were estimated (Eqs. 15–17). Mean values of tree height (m), DBH (cm), all indices, RF, and RD were compared among three different sites.

#### Statistical analyses and modelling work

We run Kolmogorov–Smirnov (K–S) Test and found the data were normally distributed. Therefore, one-way analysis of variance (ANOVA) and Tukey’s post hoc test were used to determine whether there are any statistically significant differences (p ≤ 0.05) between the three homestead forest sites and which site significantly differed from the other sites in tree biomass (Mg C ha^−1^), height (m), DBH (cm), density (individual ha^−1^), basal area, BA (m^2^ ha^−1^), Margalef richness index and Shannon–Wiener diversity index. Moreover, two-way analysis of variance (ANOVA) was performed to determine whether there are any statistically significant differences (p ≤ 0.05) of soil carbon stock (Mg C ha^−1^) against three sites and three soil depths.

Relationship between tree biomass carbon stock (Mg C ha^−1^) and tree (a) height (m), (b) DBH (cm), (c) density (individual ha^−1^), (d) basal area, BA (m^2^ ha^−1^), (e) Margalef richness index, and (f) Shannon–Wiener diversity index were modelled by using linear regression analysis. In addition, multiple regression analysis was used to model the effect of all variables (a–f) to tree biomass. The regression analyses were performed to determine the what independent variables contribute to the explanation of the biomass carbon stock and to what degree. All these statistical analyses were performed by using Statistical Package for the Social Sciences (SPSS) version 26.

## Results

### Stand structure and tree species diversity in homestead forests

**Fig. 2:**
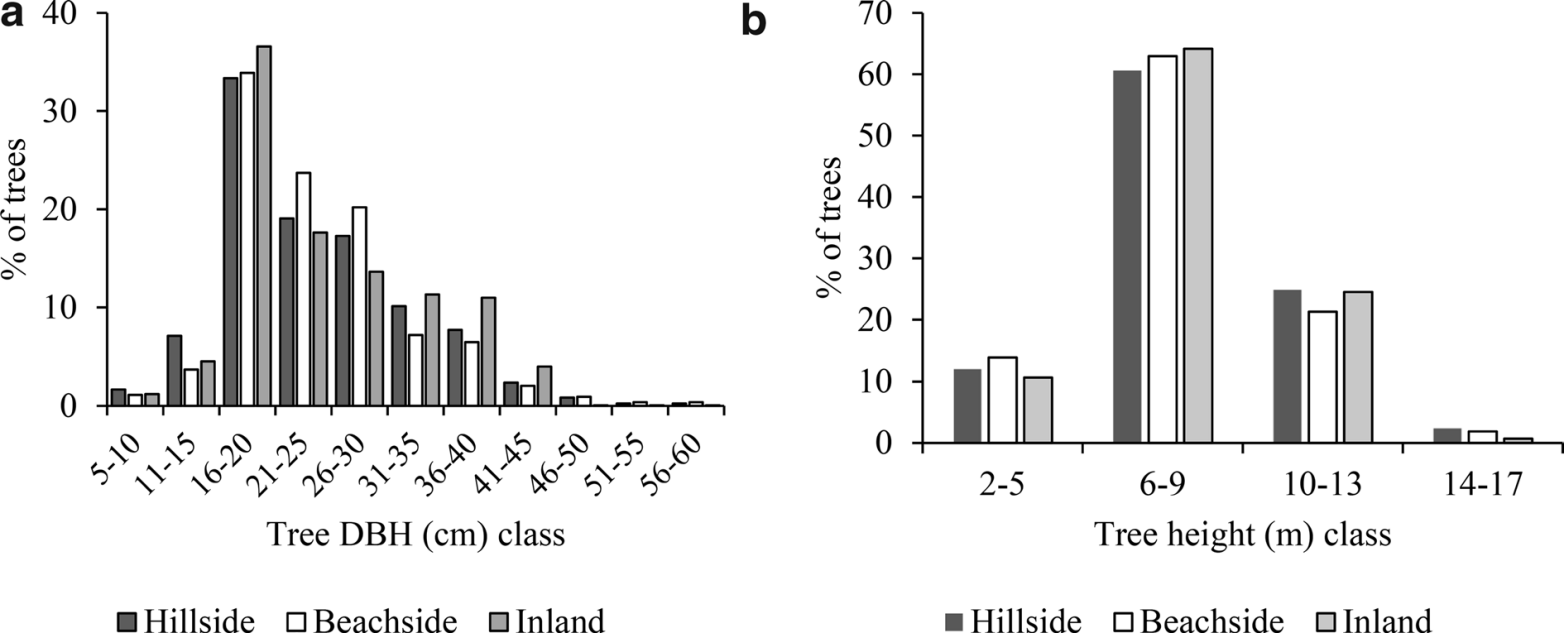
Tree (**a**) DBH (cm) and (**b**) height (m) classes in homestead forests

We found the greatest mean tree DBH and height in the hillside and lowest in the beachside homestead forests, with significant (p ≤ 0.05) differences among the three sites (Table 2). The greatest number of trees were in 16–20 cm DBH class, with the inland homestead forests site being dominated. The trees with large DBH (31–45 cm) were greater in the inland compared to the other two sites (Fig. 2a). However, there were only few trees with DBH < 15 cm due to the harvesting of those at pole stage to be used as fuels. Related to height, the greatest number of trees were in 6–9 m class. Taller trees (10–13 m) were greater in inland and hillside homestead forests (Fig. 2b). The tree species diversity and richness of homestead forests were significantly (p ≤ 0.05) greater in the hillside and inland, compared to those on the beachside (Table 2).

**Table 2:** Mean values of tree DBH, height, species diversity and richness indices in the homestead forests

Variables/site categories	Hillside	Beachside	Inland	Total mean
Mean DBH (cm)	22.87 ± 0.70^a^	14.64 ± 1.43^c^	18.98 ± 0.86^b^	18.82 ± 0.62
Mean height (m)	7.86 ± 0.22^a^	4.91 ± 0.47^c^	6.30 ± 0.28^b^	6.34 ± 0.20
Shannon–Weiner diversity index	1.44 ± 0.04^a^	0.96 ± 0.10^b^	1.29 ± 0.06^a^	1.23 ± 0.04
Margalef richness index	1.61 ± 0.06^a^	1.11 ± 0.12^b^	1.27 ± 0.07^a^	1.32 ± 0.05

Different letters within a row indicate significant differences at p ≤ 0.05 in post hoc (Tukey’s test)

**Fig. 3:**
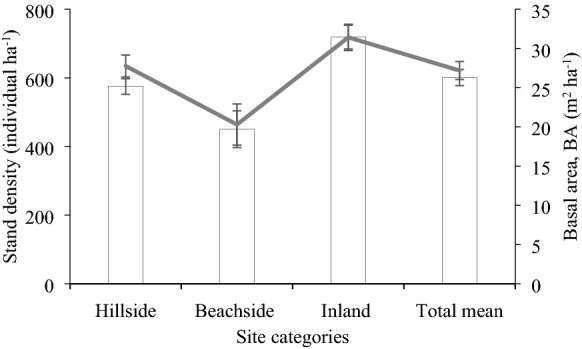
Stand density (primary y axis) and basal area (secondary y axis) in the homestead forests. Bars represent standard error of mean

Mean stand density and BA of homestead forests were 601 individuals ha^−1^ and 27 m^2^ ha^−1^, respectively, across the study area. Regarding the site, stand density in the inland homestead forests was significantly (p ≤ 0.05) greatest, compared to that in the other two sites, while no significant difference in these two (Fig. 3). BA of homestead forests was significantly (p ≤ 0.05) greater in the inland and hillside, compared to that of other one (Fig. 3).

Among 52 tree species found in the homestead forests, the numbers of species in the hillside, beachside, and inland were 41, 42, 48, respectively (Table 3). The number of tree individuals sampled were 840, 540, and 1504, respectively. The most five frequent species across the area were *Mangifera indica, Acacia auriculiformis, Cocos nucifera*, *Artocarpus heterophyllus*, and *Samanea saman* and these also corresponded to the RD (Table 3)*.*

**Table 3:** Relative frequency (RF, %) and relative density (RD, %) of species found in the homestead forests

No.	Species	RF (%)			RD (%)		
		Hillside	Beachside	Inland	Hillside	Beachside	Inland
1	*Acacia auriculiformis*	11.78	9.62	11.76	14.17	14.26	15.03
2	*Acacia mangium*	7.29	3.83	5.74	7.88	5.44	7.11
3	*Aegle marmelos*	0.30	0.43	0.00	0.24	0.19	0.00
4	*Albizia procera*	0.61	2.98	1.99	0.48	1.94	1.54
5	*Anacardium occidentale*	0.91	0.85	0.88	0.48	0.58	0.47
6	*Annona squamosa*	0.30	0.00	0.22	0.12	0.00	0.13
7	*Areca catechu*	4.56	2.55	3.31	9.07	5.24	6.24
8	*Artocarpus heterophyllus*	8.81	6.38	7.51	8.95	6.41	6.85
9	*Averrhoa bilimbi*	1.22	0.43	0.66	0.48	0.39	0.27
10	*Averrhoa carambola*	0.61	0.00	0.44	0.48	0.00	0.20
11	*Azadirachta indica*	0.00	0.43	0.22	0.00	0.39	0.20
12	*Bombax ceiba*	0.61	0.00	0.44	0.24	0.00	0.13
13	*Chukrasia tabularis*	0.61	0.00	0.66	0.60	0.00	0.27
14	*Citrus maxima*	0.91	0.85	1.32	0.36	0.58	0.74
15	*Clerodendrum viscosum*	0.30	0.43	0.22	0.12	0.19	0.13
16	*Cocos nucifera*	11.18	17.31	11.33	15.37	12.86	15.96
17	*Dellenia indica*	0.00	0.85	0.44	0.00	0.58	0.27
18	*Diospyros blancoi*	0.00	1.28	0.44	0.00	0.58	0.27
19	*Diptercarpus turbinatus*	0.91	4.26	2.43	0.36	2.33	1.54
20	*Elaeocarpus floribundus*	0.00	0.85	0.44	0.00	0.58	0.20
21	*Erythrina orientalis*	0.30	0.43	0.22	0.12	0.19	0.07
22	*Eucalyptus sp*	0.30	0.85	0.44	0.24	0.97	0.40
23	*Ficus racemosa*	0.30	0.85	0.44	0.12	0.39	0.20
24	*Garcinia cowa*	0.00	0.43	0.22	0.00	0.19	0.07
25	*Gemlina arborea*	0.91	4.26	1.99	0.48	3.50	1.81
26	*Hevea brasiliensis*	0.91	0.43	0.88	0.36	0.58	0.47
27	*Hopea odorata*	0.61	1.28	1.10	0.36	0.97	0.67
28	*Lagerstroemia speciosa*	0.00	0.00	0.44	0.00	0.00	0.20
29	*Lannea coromandelica*	0.91	1.28	1.77	0.36	0.78	1.54
30	*Lichi chinensis*	1.22	0.85	1.32	0.60	0.97	0.81
31	*Magnolia champaca*	0.61	0.00	0.22	0.24	0.00	0.07
32	*Mangifera indica*	14.59	11.06	13.02	20.41	14.76	14.16
33	*Manilkara zapota*	0.30	0.43	0.66	0.12	0.39	0.34
34	*Mimusops elengi*	0.00	0.43	0.22	0.00	0.39	0.13
35	*Moringa oleifera*	0.30	0.43	0.00	0.12	0.19	0.00
36	*Neolamarckia cadamba*	0.30	0.43	0.22	0.12	0.39	0.27
37	*Phoenix dactylifera*	0.61	0.00	0.44	0.24	0.00	0.27
38	*Phyllanthus emblica*	0.00	0.43	0.22	0.00	0.19	0.20
39	*Polyalthia longifolia*	0.30	0.00	0.44	0.12	0.00	0.20
40	*Psidium guajava*	4.56	5.11	3.97	4.06	3.88	3.56
41	*Pterygota alata*	0.61	0.43	0.44	0.24	0.19	0.34
42	*Samanea saman*	6.08	8.09	7.06	4.53	5.24	5.84
43	*Spondias pinnata*	0.61	0.85	0.22	0.24	0.78	0.07
44	*Swietenia mahagoni*	5.17	7.66	5.96	4.65	7.96	5.44
45	*Syzygium samarangense*	0.00	0.43	0.00	0.00	0.19	0.00
46	*Syzygium sp*	3.65	2.13	2.87	1.91	1.36	2.08
47	*Tamarindus indica*	0.00	0.43	0.44	0.00	0.19	0.34
48	*Terminalia arjuna*	0.61	0.43	0.44	0.24	0.19	0.40
49	*Terminalia catappa*	0.00	0.00	0.44	0.00	0.00	0.27
50	*Trewia nudiflora*	0.61	0.00	0.00	0.36	0.00	0.00
51	*Vitex peduncularis*	0.61	0.85	0.88	0.24	0.58	0.94
52	*Ziziphus mauritiana*	4.56	4.26	3.53	2.86	2.91	1.95

### Tree (above-and below-ground) biomass and litterfall carbon in homestead forests

**Fig. 4:**
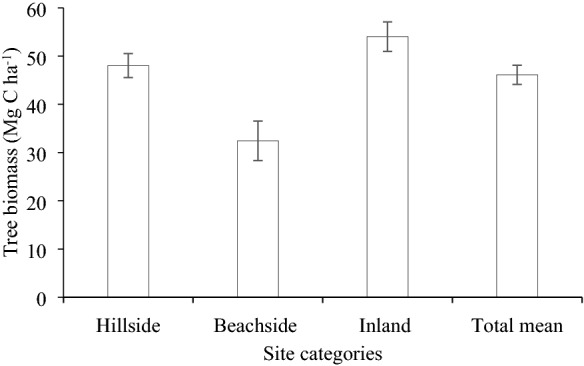
Tree (above-and below-ground) biomass in the homestead forests. Bars represent standard error of mean

Mean tree (above-and below-ground) biomass in the homestead forests was estimated to be 46.11 Mg C ha^−1^ across the study area. Tree biomass was significantly (p ≤ 0.05) greater in the hillside and inland, compared to that in the beachside homestead forests (Fig. 4). The mean dry biomass of the litterfall was 0.04 ±  0.01 Mg C ha^−1^ across the study area. These were 0.06 ± 0.02,  0.04 ± 0.01, and 0.03 ± 0.02  Mg C ha^−1^ in the hillside, beachside and inland, respectively.

**Fig. 5:**
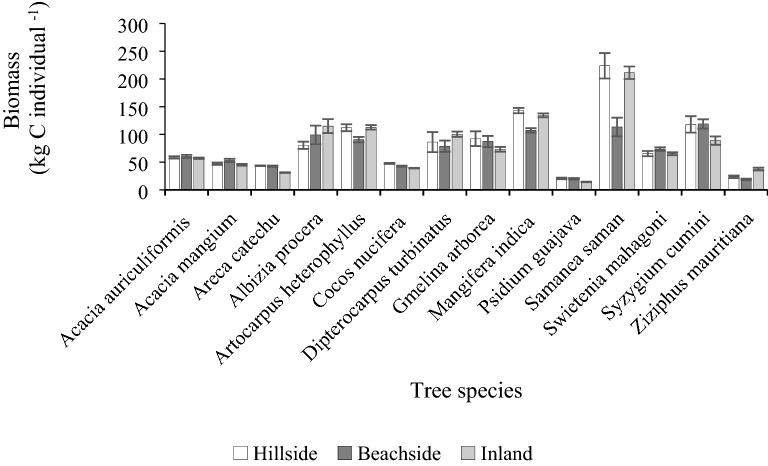
Tree (above-and below-ground) biomass for most frequent species in the homestead forests. Bars represent standard error of mean

Among the species, *Samanea saman* dominated in storing carbon, with *Mangifera indica*, *Artocarpus heterophyllus*, *Diptercarpus turbinatus*, and *Albizia procera* stored relatively greater amount of biomass carbon in the inland and hillside homestead forests, compared to the beachside forest (Fig. 5).

### Carbon concentration and stocks in mineral soil

**Fig. 6:**
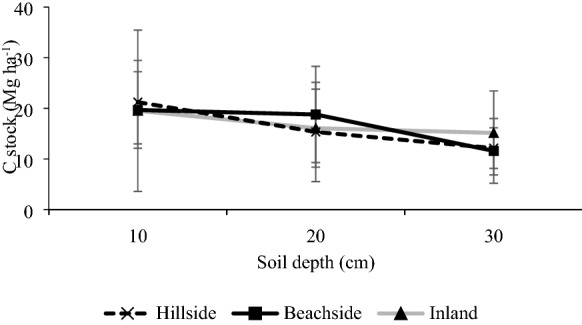
Carbon stocks in mineral soil at three different depths in homestead forests. Bars represent standard error of mean

Carbon concentration and stocks diminished with increasing depth of soil in homestead forests across three sites (Table 4; Fig. 6). The greatest mean carbon stocks were found throughout the soil depths in the inland homestead forests. However, carbon stocks did not significantly (p ≤ 0.05) vary with sites and with soil depths (Fig. 6). The bulk density of soils increased with depth for all sites (Table 4).

**Table 4:** Soil carbon (C) concentration and bulk density (BD) at three different depths in the homestead forests

Site categories	Soil depth (cm)	BD (g cm^−3^)	C concentration (mg g^−1^)
Hillside	10	1.3	16.33
	20	1.31	5.85
	30	1.35	3.00
Beachside	10	1.3	15.14
	20	1.38	6.81
	30	1.4	2.76
Inland	10	1.31	14.90
	20	1.32	6.09
	30	1.36	3.71

### Relationship of tree biomass with structural compositions in homestead forests

**Fig. 7:**
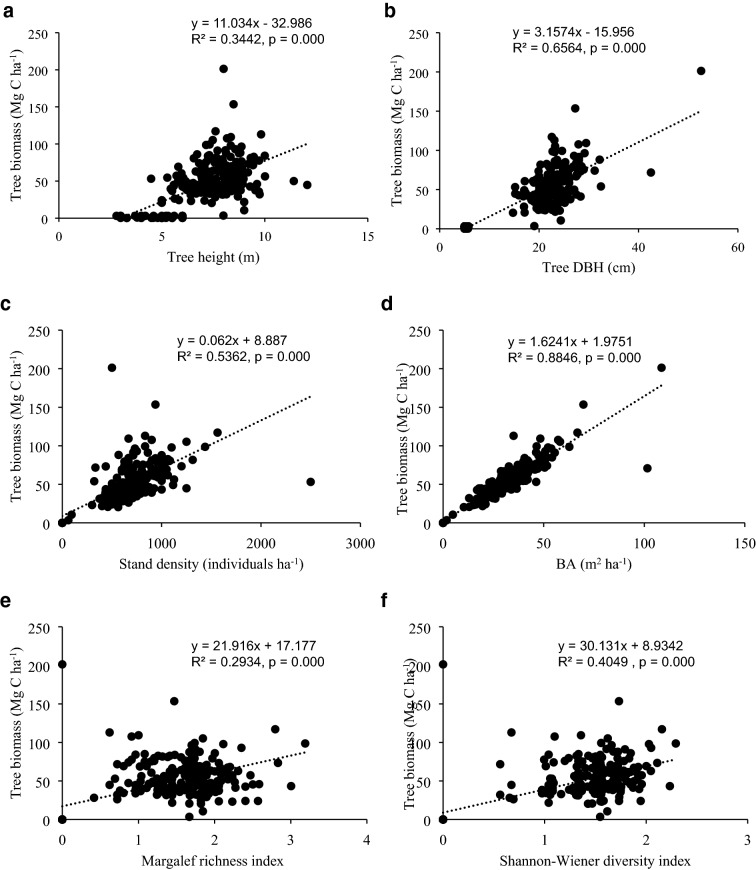
Relationship between tree biomass and (**a**) height, (**b**) DBH, (**c**) stand density, (**d**) basal area, (**e**) Margalef index, and (**f**) Shannon–Wiener index in the homestead forests

Figure 7 shows the significant (p ≤ 0.05) relationship between tree biomass (Mg C ha^−1^) and height (m), DBH (cm), density (individual ha^−1^), basal area, BA (m^2^ ha^−1^), Margalef richness index, and Shannon–Wiener diversity index in the homestead forests across three sites. Multiple regression analysis reveald that 90% of the variability in biomass C was explained by these factors together.

## Discussion

Taking urgent action to combat climate change and its impacts are amongst the Sustainable Development Goal 13, which provides us with a common plan and agenda to tackle climate change [92]. Storing carbon in a forest ecosystem helps in removing CO_2_ emissions from the atmosphere and thus, contributing to the climate change mitigation. This requires growing trees in and outside the large-scale forests, for instance in homestead forests. Realizing this potential, this study estimated the carbon stocks in the homestead forest ecosystems in an Upazila of Maheshkhali island, a hilly and coastal area in Southern Bangladesh, and estimated how tree species diversity and stand structural variation affect these carbon stocks.

Mean biomass carbon stock estimated (46 Mg C ha^−1^) in our case was close to that found (54 Mg C ha^−1^) in homestead forests in northern Bangladesh [17]. This, however, is lower than that found in the mangrove (99 Mg C ha^−1^; [22]) and total forests (49–121 Mg C ha^−1^; [12]) of Bangladesh. This disagreement could be explained by the lower overall species diversity and richness in our study, which indicates lower biomass C stock [15]. For example, Nath et al. [18] estimated tree biomass of 118 Mg C ha^−1^, with a diversity index of 2.21 in homestead forests, whereas in our case the index was 1.24. However, we found the significantly positive effects of tree species diversity and richness on biomass carbon stock. Greater species richness and diversity index in the inland and hillside homestead forests indicated higher above- and below-ground biomass carbon stocks compared to that in the beachside. An increase in species richness and diversity index by one unit increased the biomass carbon stock by 22 and 30 Mg C ha^−1^, respectively (Fig. 7e, f). Our findings agreed with earlier studies in tropical forests of Asia and Africa [29, 30, 93], implying the more tree species diversity and richness the more likely higher above-ground biomass carbon stock.

Our study revealed a greater contribution of some of the frequently occurred species to the total carbon stocks. The relatively greater number of individuals of *Mangifera indica*, *Samanea saman*, *Artocarpus heterophyllus*, and *Dipterocarpus turbinatus* in the inland and hillside homestead forests, contributed to the greater carbon stocks compared to the beachside. Alamgir and Al-Amin [26] also found a greater biomass carbon stock in these tree species in the hill forests of Bangladesh. The strongly positive effects of BA and stand density on biomass carbon stocks are also generally in line with the findings of [94] who reported that greater density (4258 trees ha^−1^) and BA (53 m^2^ ha^−1^) increased biomass carbon stocks in roadside plantations.

In this study, with greater DBH and height, above- and below-ground biomass carbon stocks were 48–67% greater in inland and hillside homestead forests than in beachside forests. The overall share of individuals with DBH of 31–40 cm was also greater in the hillside and inland homestead forests which contributed to the greater above- and below-ground biomass carbon stock in comparison with that of the beachside. In our case, when tree height and DBH increased by one unit each, the biomass carbon stock increased by 11 and 3 Mg C ha^−1^, respectively (Fig. 7a, b). The importance of contribution of larger trees to the biomass C stock is in line with [24] who depicted that individuals with DBH of 10–56 cm, constituting only 28% of stand density, contributed 84% of the total biomass carbon stock in mangrove forest.

The carbon stocks of tropical litterfall have not received much attention in research as it constitutes a small fraction of above-ground biomass [32, 95]. The overall carbon stored in litterfall was 0.1% of the total biomass C in this homestead forest, while it was 1.8% in the natural forests of Bangladesh [34]. The carbon stock in litterfall was greatest in the hillside, which was up to 53–83% greater than that in the beachside and inland homestead forests. Litter accumulates in natural forests as they are no longer under silvicultural management due to the harvesting restriction, while litters in homestead forests are used as cooking fuel [96, 97], which leads to lower C stocks.

There was a clear decline in soil carbon concentration and stocks across three homestead forest sites. Total soil carbon stock in the inland homestead forests across the three depths was greatest (51 Mg ha^−1^), with increased stand density and species richness. The greater carbon in soil was correlated with greater stand density, and species diversity and richness, which has been found earlier in tropical agroforestry systems [46, 52] and temperate forests [43, 98]. The surface soil had 5–38% and 29–75% greater carbon stock of that stored at a depth of 20 and 30 cm, respectively, depending on the site. Hillside forests with greater litterfall, had 8–9% greater surface soil carbon stock compared to beachside and inland forests. *Acacia auriculiformis*, *Acacia mangium*, and *Swietenia mahagoni* species were abundant in the hillside and inland forests, contributing to soil C stock. This was because, Acacia and Mahagani litters were not preferred as fodder or fuels due to being small leaflet and unpalatable, contrary to *Mangifera indica* and *Artocarpus heterophyllus* [35]. Acacia species planted site in Bangladesh and African Mahogani in Ghana were found enhancing soil carbon stock [35, 41, 99]. Earlier studies on homestead forest and agroforestry in India also reported that the slower decay rate of Acacia and *S. mahagoni* litters resulted in accumulation of organic matters in the soil, compared to *M. indica, A. heterophyllus and Anacardium occidentale* [44, 100].

The relationships found between both the stand structure and tree species diversity with the biomass carbon have some important implications for emission reduction under the REDD and its derivative REDD + programme. The homestead forests with high floral diversity and biomass carbon indicate their high conservation potential. Therefore, carbon storing in homestead forests can provide co-benefits of biodiversity conservation under the REDD programme as it aims to maintain carbon stock in tropical forests while protecting threatened tree species [55, 58]. In this study, *Garcinia cowa* and *Vitex peduncularis* appeared as rare species [101] and can be conserved by protecting them from further erosion. While state-owned forests decline in Bangladesh, reducing carbon stocks and biodiversity in forests, the biodiverse-rich homestead forests are in a crucial role in enhancing carbon sinks, reducing emissions from deforestation, and contributing to global carbon cycle. These forests are managed for variable household livelihood options, which could provide carbon credits under REDD + programme. An appropriate management in community forests in Nepal have contributed to REDD + and local livelihoods [102–104], for example. However, a regulatory framework would be required to take homestead or small-scale forests under REDD and REDD + programme for policy initiatives to safeguard carbon, biodiversity, and local livelihood.

At local level, stand structural traits (DBH, height, BA, density) can easily be measured in the field by homestead forest owners or local communities. Based on these field data from small spatial scale, one can also assess biomass or produce map over large area using remote sensing techniques, and estimate national carbon storage and deforestation in TOF for REDD + monitoring.

## Conclusions and policy implications

The hillside and inland homestead forests stored remarkable amount of tree biomass carbon, which was significantly increased with increasing stand density, BA, DBH, height, species richness and diversity. A smaller carbon stocks in litter found in our study compared to earlier studies could be linked to the removal of litter for using it as fuels. Higher litterfall in the hillside homestead forests may have contributed to surface soil carbon stocks, but the overall soil carbon stock in the study was also affected by types of litter of species, the stand density, and species richness. However, the decay of litter and humus and underground process in tropical forests influencing soil carbon stock, depending on environmental factors, would need to be studied further.

In Bangladesh, total annual emissions of the energy sector are 78 Mt of CO_2_ [9]. Conversely, the land use and land use change and forestry (LULUCF) sector’s carbon sink was 81 Mt in 2010 [105]. According to our study, the homestead forest ecosystems (trees, litterfall and soil) store 96 Mg C ha^−1^, which is 73 and 62% of that found in mangrove and hill forests (133 and 154 Mg C ha^−1^, respectively [16, 22]. This can be upscaled to be 26 Mt considering total homestead forest area of the country, which thus can contribute to climate change mitigation through REDD + and CDM mechanisms as emphasized in UNFCCC’s mitigation strategies [2, 106].

This study reduced the gap with documentation and producing estimated carbon of homestead forests that would help for applying REDD + mechanism since the contribution of TOF in carbon sequestration is ignored due to the scarce documentation [62]. In addition, the empirical and analytical results of this study could be a source of carbon credits through Payment for Environmental Services for small-scale homestead forests owners or households in developing countries, such as Bangladesh, while generating livelihood options and biodiversity conservation [61, 62].

## Data Availability

All data generated or analysed during this study are included in this article.

## Abbreviations

AGB: Above ground biomass
BGB: Below ground biomass
BA: Basal area
BD: Bulk density
C: Carbon
DBH: Diameter at breast height
LOI: Loss of ignition
RF: Relative frequency
RD: Relative density
SOC: Soil organic carbon
TB: Total biomass
TOF: Tree outside forest

## Footnotes

1 The second lowest administrative and management unit at the field level of Bangladesh Forest Department, BFD.

2 The second lowest administrative and management unit at the local level of the governing system of Bangladesh.
